# Ecophysiological, morphological, and biochemical traits of free-living *Diplosphaera chodatii* (Trebouxiophyceae) reveal adaptation to harsh environmental conditions

**DOI:** 10.1007/s00709-021-01620-6

**Published:** 2021-02-07

**Authors:** Cynthia Medwed, Andreas Holzinger, Stefanie Hofer, Anja Hartmann, Dirk Michalik, Karin Glaser, Ulf Karsten

**Affiliations:** 1 Institute of Biological Science, Applied Ecology & Phycology, University of Rostock, Albert-Einstein-Strasse 3, D-18059 Rostock, Germany; 2 Department of Botany, Functional Plant Biology, University of Innsbruck, Sternwartestrasse 15, A-6020 Innsbruck, Austria; 3 Department of Pharmacognosy, University of Innsbruck, Innrain 80-82, A-6020 Innsbruck, Austria; 4 Institute of Chemistry, University of Rostock, Albert-Einstein-Strasse 3a, D-18059 Rostock, Germany; 5 Leibniz Institute of Catalysis, Albert-Einstein-Strasse 29a, D-18059 Rostock, Germany

**Keywords:** Aeroterrestrial microalgae, Desiccation, Growth rates, Photosynthetic irradiance curve, MAAs, Polyols

## Abstract

Single-celled green algae within the Trebouxiophyceae (Chlorophyta) are typical components of terrestrial habitats, which often exhibit harsh environmental conditions for these microorganisms. This study provides a detailed overview of the ecophysiological, biochemical, and ultrastructural traits of an alga living on tree bark. The alga was isolated from a cypress tree in the Botanical Garden of Innsbruck (Austria) and identified by morphology and molecular phylogeny as *Diplosphaera chodatii*. Transmission electron microscopy after high-pressure freezing (HPF) showed an excellent preservation of the ultrastructure. The cell wall was bilayered with a smooth inner layer and an outer layer of polysaccharides with a fuzzy hair-like appearance that could possibly act as cell-cell adhesion mechanism and hence as a structural precursor supporting biofilm formation together with the mucilage observed occasionally. The photosynthetic-irradiance curves of *D. chodatii* indicated low light requirements without photoinhibition at high photon flux densities (1580 μmol photons m^−2^ s^−1^) supported by growth rate measurements. *D. chodatii* showed a high desiccation tolerance, as 85% of its initial value was recovered after controlled desiccation at a relative humidity of ~10%. The alga contained the low molecular weight carbohydrates sucrose and sorbitol, which probably act as protective compounds against desiccation. In addition, a new but chemically not elucidated mycosporine-like amino acid was detected with a molecular mass of 332 g mol^−1^ and an absorption maximum of 324 nm. The presented data provide various traits which contribute to a better understanding of the adaptive mechanisms of *D. chodatii* to terrestrial habitats.

**Supplementary Information:**

The online version contains supplementary material available at 10.1007/s00709-021-01620-6.

## Introduction

Over millions of years in the evolutionary history of photosynthetic eukaryotic microalgae, these organisms established themselves successfully on land (Vries and Archibald 2018). There, terrestrial microalgae perform important, multifunctional ecological roles, for example, in primary production, stabilization of soils, and nitrogen cycling (Karsten and Holzinger 2014).

Terrestrial microalgae occur in all climatic regions and can be found in various habitats, such as walls of urban buildings (Raabová et al. 2016), in biological soil crusts in alpine regions and elsewhere (Lewis and Flechtner 2002; Karsten and Holzinger 2014), or as Antarctic endoliths (Büdel et al. 2016). Furthermore, several unicellular terrestrial green microalgae like members of the genera *Stichococcus* and *Diplosphaera* can act as typical photobionts in lichens (Fontaine et al. 2012). Independent of the occurrence in biological soil crusts, biofilms, or in symbiosis, terrestrial microalgae are typically exposed to a wide range of rapidly changing environmental parameters.

Frequent fluctuations of environmental conditions are common in most terrestrial habitats, in contrast to rather stable aquatic habitats. Most important stressors for terrestrial green algae are desiccation, high temperatures, and solar radiation. Terrestrial algae developed specific morphological, physiological, and biochemical acclimation mechanisms to cope with extreme environmental stressors (Hoffmann 1989), such as the capability to synthesize water-holding osmolytes (Yancey 2005) and UV sunscreens (Hartmann et al. 2020) or to form colonies and biofilms which also reduce uncontrolled water loss (Karsten et al. 2007a).

Ultraviolet radiation (UVR), as a natural fraction of solar radiation, negatively influences life in aquatic ecosystems (Karsten 2008) but for many phototrophs on land UV-A and UV-B are considered major stress factors, too. UVR exerts many harmful effects on metabolism and can cause DNA damage. Apart from molecular damages, inhibition of photosynthetic primary productivity incurs a fundamental problem for phototrophic organisms. For this reason, various terrestrial representatives of the green algal class Trebouxiophyceae developed the capability to synthesize UV-absorbing mycosporine-like amino acids (MAAs) that are widely distributed among marine organisms too (Bandaranayake 1998). MAAs are low-molecular weight compounds with numerous chemical variations in their side groups and substituents, resulting in different absorption maxima between 310 and 360 nm (Cockell and Knowland 1999; Singh et al. 2008). MAAs shield the cell from UVR by absorbing the radiation energy and converting it into harmless heat, without generating photochemical reactions which are known to be harmful for cells (Bandaranayake 1998). Thus, biosynthesis and accumulation of these UV-absorbing sunscreens are an essential key protective mechanism for these terrestrial microalgae. Investigations on unstudied terrestrial microalgae from harsh environments have the potential to discover new MAAs, such as the recently described klebsormidin A and B from the streptophytes *Klebsormidium* and *Interfilum* (Hartmann et al. 2020).

Terrestrial microalgae, especially such as *Apatococcus*, *Prasiola*, and *Stichococcus* (Trebouxiophyceae), are protected against desiccation by synthesizing and accumulating low-molecular weight carbohydrates (LMWCs), such as the polyols glycerol, ribitol, mannitol, and sorbitol (Gustavs et al. 2011; Hotter et al. 2018). These organic osmoprotectants are considered effective stress metabolites (Gustavs et al. 2011), and the polyol metabolism forms a fundamental part of the biochemical protective mechanism against desiccation. With the accumulation of LMWCs, terrestrial microalgae maintain turgor, membrane, and macromolecule structure by compensating differences in water potential (Farrant 2000; Fernández-Marín et al. 2016), and many of these compounds act additionally as compatible solutes (Yancey 2005; Holzinger and Karsten 2013). Polyols can also act as antioxidants or heat protectants, leading to a stabilization of proteins (Karsten et al. 2007a). Furthermore, these LMWCs are used as specific chemotaxonomic markers which have been investigated several times for different terrestrial green algal taxa (Gustavs et al. 2011; Hotter et al. 2018).

Overall, living in terrestrial habitats as single or colony-forming green algae is challenging because of frequently changing environmental stressors like UVR, desiccation, and temperature. Thus, algae developed various protective mechanisms to withstand fluctuating, often harsh conditions and thereby to guarantee long-term survival. The impressive diversity of terrestrial microalgae and their diverse morphology as well as their persistence in such environments suggest that they offer excellent model systems to explore physiological tolerance and survival strategies, which have been developed during making the transition from water to land.

In this study, we focused on ecophysiological, biochemical, and ultrastructural traits of a Trebouxiophycean microalga for a better understanding of possible adaptive strategies to the harsh environment on top of a tree bark. Although at least 150 unicellular species of aeroterrestrial Chlorophyta are morphologically described (Ettl and Gärtner 2013), neither their adaptation to environmental conditions, their ultrastructural features nor their phylogenetic relationships are well understood.

## Material and methods

### Habitat, isolation, and culture conditions

**Fig. 1:**
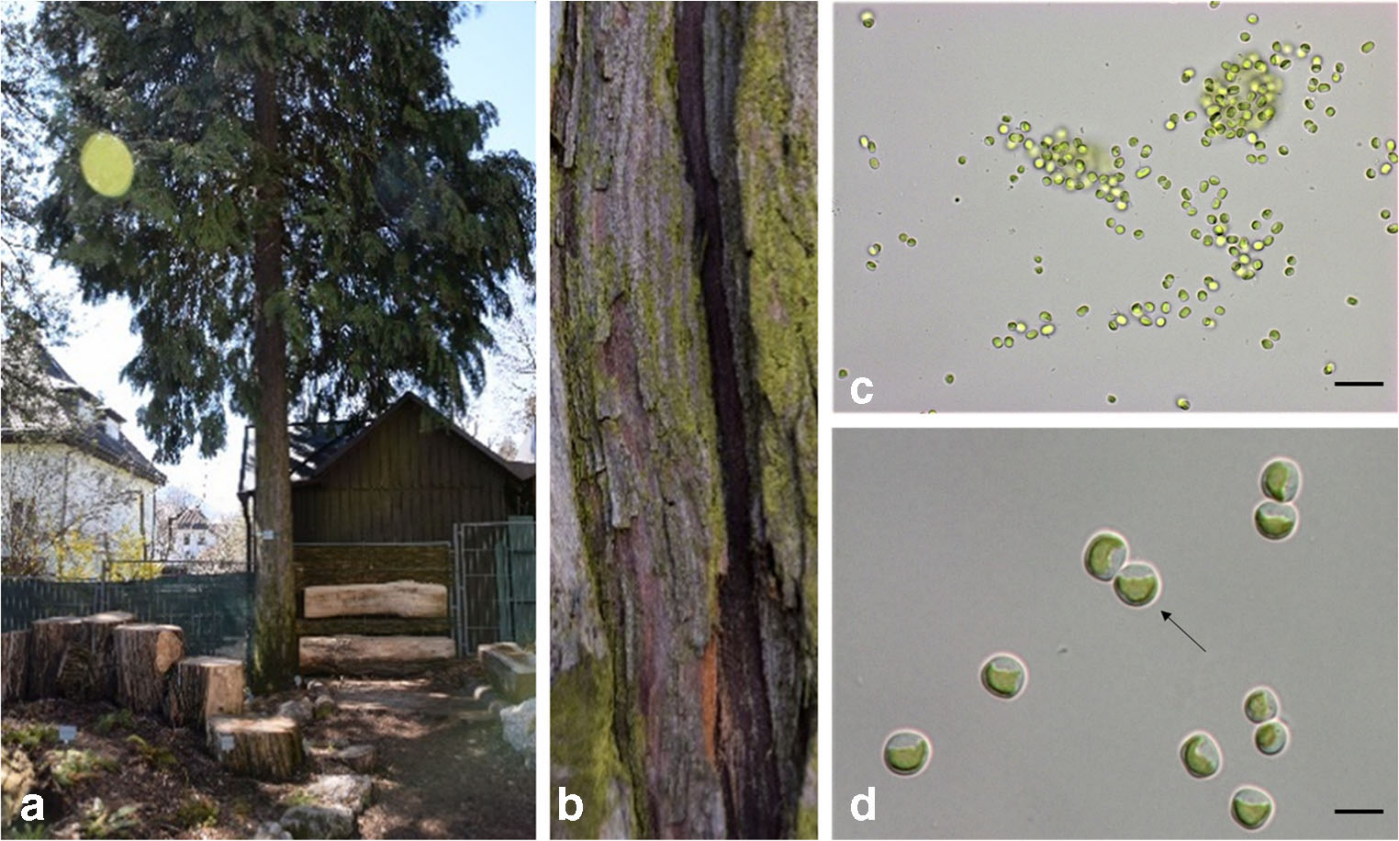
Habitat of isolated terrestrial green alga and micrographs of log-phase *Diplosphaera chodatii* CM01 (Trebouxiophyceae). **a** Cypress tree (*Chamaecyparis lawsoniana)*, **b** bark with algal biofilm, **c** singular and dyads formed isolate CM01 in liquid culture, variation in cell shape, **d** cup-shaped parietal chloroplast (arrow), scale bars **c** 20 μm, **d** 5 μm

*Diplosphaera chodatii* was isolated from bark of a cypress tree (*Chamaecypris lawsoniana*) in the Botanical Garden of Innsbruck (Austria) at 616 m a.s.l. (47° 16′ 4″ N, 11° 22′ 42″ E) (Fig. 1a, b). The strain was isolated in May 2017, purified and established as clonal culture by a procedure described by Tschaikner (2008). During the day, the tree is mainly shaded except in the morning hours when about 50% of the bark receives sunlight. *Diplosphaera chodatii* was cultivated as strain CM01 in Erlenmeyer flasks (volume 250–500 mL) filled with modified Bold’s Basal Medium (Starr and Zeikus 1993). The cultures were kept at 40 to 50 μmol photons m^−2^ s^−1^ (Lumilux Deluxe Daylight L15W/950; OSRAM) in a 16 h-light-8 h-dark cycle at 20 °C.

### Sequencing and morphological characterization

For sequencing of the unicellular bark alga (isolate CM01), DNA was extracted from 8 mg lyophilized dry extract using the NucleoSpin Plant II kit (MACHEREY-NAGEL GmbH & Co. KG, Germany). Phylogenetic analysis was implemented using the genetic marker *rbc*L (large subunit of the ribulose bisphosphate carboxylase/oxygenase RuBisCO). The *rbc*L gene was chosen as genetic marker as Rindi et al. (2007) already demonstrated that this gene region seems to show a higher sequence divergence within the Prasiolales than the more conserved 18S ribosomal RNA gene. The *rbc*L gene region was amplified and sequenced using the primers: SH F5 and SH R8 according to Heesch et al. (2012). Sequencing was performed by Eurofins GfA GmbH (Germany) quality checked and assembled using Chromas 2.6. Sequences were aligned manually in MEGA X (version 10) (for more details, see Kumar et al. (2018)). The sequence is stored at GenBank under the accession number MT921816.

For morphological characterization, light microscopy was performed using a Zeiss Axiovert 200 M; micrographs were captured with a Zeiss Axiocam MRc5 (Carl Zeiss AG, Germany). For ultrastructural characterization, transmission electron microscopy (TEM) was performed after a high pressure freezing (HPF) followed by freeze substation (FS) according to previously published protocols (Aichinger and Lütz-Meindl 2005). *Diplosphaera chodatii* cells were fixed with a LEICA EMPACT high-pressure freezer and freeze substituted in a Leica EM AFS FS apparatus (Leica Microsystems, Austria). Freeze substitution was performed in 2% OsO_4_ and 0.05% uranyl acetate in acetone at − 80 °C for 60 h; temperature was raised to − 30 °C within 5 h (10 °C h^−1^), maintained at − 30 °C for 4 h; then, temperature was raised to 20 °C within 20 h (2.5 °C h^−1^). Samples were embedded in Agar Low viscosity resin kit (Agar Scientific, England). Ultrathin sections (~60 nm) were prepared with a Reichert Ultracut (Leica AG, Austria), stained with 2% uranyl acetate (10 min) and Reynold’s lead citrate (2 min), and viewed with a ZEISS Libra 120 TEM (Zeiss, Germany) at 80 kV.

### Photosynthesis measurements with an oxygen optode

Photosynthetic oxygen evolution rates of *D. chodatii* were measured under increasing photon flux densities from 0 to 1580 μmol photons m^−2^ s^−1^ according to Karsten et al. (2010b) and Prelle et al. (2019). Presens Fibox3 oxygen optodes (Presens, Germany) and 4 × 3 mL thermostatic acrylic chambers DW1 (Hansatech Instruments, UK) combined with magnetic stirrers were used at an ambient temperature of 20 °C. The O_2_ production per photon flux density and time was normalized to the amount of total chlorophyll *a* per sample. After photosynthesis-irradiance (P-I) curve measurements, the cell suspension was filtered onto Whatman GF/F glass fiber filters (Whatman, Dassel, Germany). Chlorophyll *a* was extracted with 90% aqueous ethanol (v/v) overnight at 4 °C and quantified according to Ritchie (2008). P-I curve data were calculated and fitted by the mathematical photosynthesis model of Walsby (1997) with the SOLVER-function from Excel (Microsoft Office 365) which allowed the calculation of the three parameters: α (positive slope at limiting photon flux densities), I_C_ (light compensation point), and I_K_ (initial value of light-saturated photosynthesis).

### Growth experiments depending on light using a growth fluorimeter

The growth rate of *D. chodatii* CM01 under different light conditions was recorded by daily measuring the chlorophyll *a* fluorescence with a growth fluorimeter (Hansatech, UK) according to Karsten et al. (1996). Before the experiment started, *D. chodatii* was acclimated to the respective experimental conditions for 5 days. Afterwards, 100 μL was taken from each acclimatized pre-culture and transferred into disposable petri dishes with cover lids (LICEFA GmbH & Co. KG, Germany) as incubation vessels. Before each measuring day, the fluorimeter was calibrated with a glass cuvette (Schott) whose bottom was covered with a filter combination of long pass glass filter (RG 665, Schott) and a non-fluorescent red gelatin filter (for more details, see Karsten et al. (1996)). Each sample was measured three times, after an illumination period of 5 s. Before each measurement, the samples were incubated in the dark for 10 min. As proxy for growth, the chlorophyll *a* fluorescence was regularly measured every 24 h over an unusually long period of 28 days. A logistic growth model was selected to calculate the growth rates. For this purpose, the measured fluorescence per day was calculated using the following formula after Heuser (2003):

$$ {\mathrm{F}}_{\mathrm{t}}=\frac{\mathrm{K}\cdotp {\mathrm{F}}_0\cdotp \exp \left(\upmu \cdotp \left({\mathrm{t}}_{\mathrm{n}}-{\mathrm{t}}_0\right)\right)}{\mathrm{K}+{\mathrm{F}}_0\cdotp \left(\exp\ \left(\exp\ \left(\upmu \cdotp \left({\mathrm{t}}_{\mathrm{n}}-{\mathrm{t}}_0\right)-1\right)\right)\right)} $$

F_t_: calculated fluorescence
K: capacity or highest value that a population can reach in a given time (value with strongest fluorescence signal)
F_0_: initial fluorescence
μ: growth rate
t_n_: day n of calculated fluorescence
t_0_: day at time when F_0_ was measured

### Effect of controlled desiccation and rehydration on effective quantum yield of PSII

To follow the kinetics of controlled desiccation and subsequent rehydration, a standardized set-up after Karsten et al. (2014) was applied using a 200-mL polystyrol box. Different desiccants were tested to achieve different humidities (Supp. Table 1). For the final experiment, silica gel was used as desiccant to create a relative humidity (RH) of ~10%. The effective quantum yield of PSII (Y(II)) was determined every 10 to 30 min during the dehydration and rehydration period using non-invasive pulse amplitude modulation fluorometry (PAM2500, Walz, Germany), whereby the fiber optics of the light probe was kept in an equal distance to the growth chamber throughout the experiment. Four times 50 μL of the algal suspension were concentrated in four spots on Whatman GF/F glass fiber filters (Whatman, Germany). The cells were dried until the average of the effective quantum yield ceased (ca. 45 to 55 min). After the dehydration period, the fiber glass filters were transferred into a new polystyrol box filled with 100 mL tap water and rehydrated by adding 200 μL of tap water to each filter (4 × 50 μL per spot) to create a high RH (> 95%). The filters were kept under low-light (30–40 μmol photons m^−2^ s^−1^) conditions, and RH inside the polystyrol box was continuously recorded using a MSR 145W (MSR Electronics GmbH, Switzerland).

In order to compare the laboratory desiccation experiment with the field situation, a 12-cm^2^ piece of the cypress bark with an attached *D. chodatii* biofilm was taken in June 2017 at noon to measure the effective quantum yield in the air-dried state with the PAM 2500 (Walz, Germany). Afterwards, the biofilm was artificially rewetted by spraying tap water, and the recovery kinetics of the effective quantum yield followed in short intervals for the next 60–70 min.

### Screening for mycosporine-like-amino acids and osmoprotectants

*Diplosphaera chodatii* was screened for the presence of LMWCs and mycosporine-like amino acids (MAAs) using high-performance liquid chromatography (HPLC). The algal culture was transferred to glass microfiber filters (Whatman, General Electric, USA). The filters were comminuted and extracted using 10 mL of 50% aqueous methanol (v/v) in an ultrasonic bath at 30 °C for 15 min. The extracts were combined and the solvent was removed with a rotary evaporator to reach a concentration of 1 mg mL^−1^. Afterwards, samples were measured using an Agilent 1260 HPLC system (Santa Clara, CA, USA) which was coupled to an amaZon iontrap mass spectrometer (Bruker, Bremen, Germany). Water comprising 20 mM ammonium formate (A) and methanol/water (80:20) with 20 mM ammonium formate (B) served as mobile phases with a flow rate of 0.25 mL min^−1^ and an oven temperature of 30 °C. The gradient was as follows: 0–5 min 2% (B), 5–15 min from 2 to 15% (B), 15–20 min 15–50% (B), 20–22 min 50% (B), and then re-equilibrating for 8 min at 2% (B). The diode array detection ranged from 210 to 600 nm with specific wavelengths of 310, 320, 325, and 330 nm. MS spectra were recorded in positive-ESI mode (capillary voltage 4.5 kV), with a drying gas temperature of 300 °C, the nebulizer gas (nitrogen) set to 25 psi, and a nebulizer flow (nitrogen) of 12.0 L min^−1^.

NMR spectroscopy was performed to detect LMWCs. At first, 10 mg lyophilized biomass of CM01 was transferred onto a Whatman GF/F glass fiber filter (Whatman, Germany) and incubated in a 15-mL Falcon tube (Fisher Scientific GmbH, Germany) in 70% aqueous ethanol (v/v) at 70 °C for 3 h. For higher extraction success, the tubes were vortexed occasionally. After centrifugation at 13,000*g* for 5 min, 800 μL of the supernatant was transferred to a new vial and evaporated to dryness under vacuum (RVC 2-25 CD plus, Lyophylle alpha 2-4 LSC plus; Martin Christ, Germany).

The pellet was re-dissolved in 0.4 mL D_2_O (99.9%) for ^13^C-NMR spectroscopy. The NMR spectrum was recorded by a Bruker spectrometer (^1^H: 500.13 MHz; ^13^C: 125.8 MHz, AVANCE Neo 500 spectrometer, Bruker BioSpin GmbH, Germany). Chemical shifts δ are given in ppm relative to the signal for internal tetramethylsilane (TMS, δ = 0). The calibration of spectra was carried out externally, using the signals of acetone (1%, δ (^13^C) = 30.2 ppm) in D_2_O. Samples were run in 5-mm-diameter tubes at 298 K using a sweep width of 30,000 Hz and a number of 10,000 transients.

## Results

### Phylogenetic identification and morphological characteristics

**Fig. 2:**
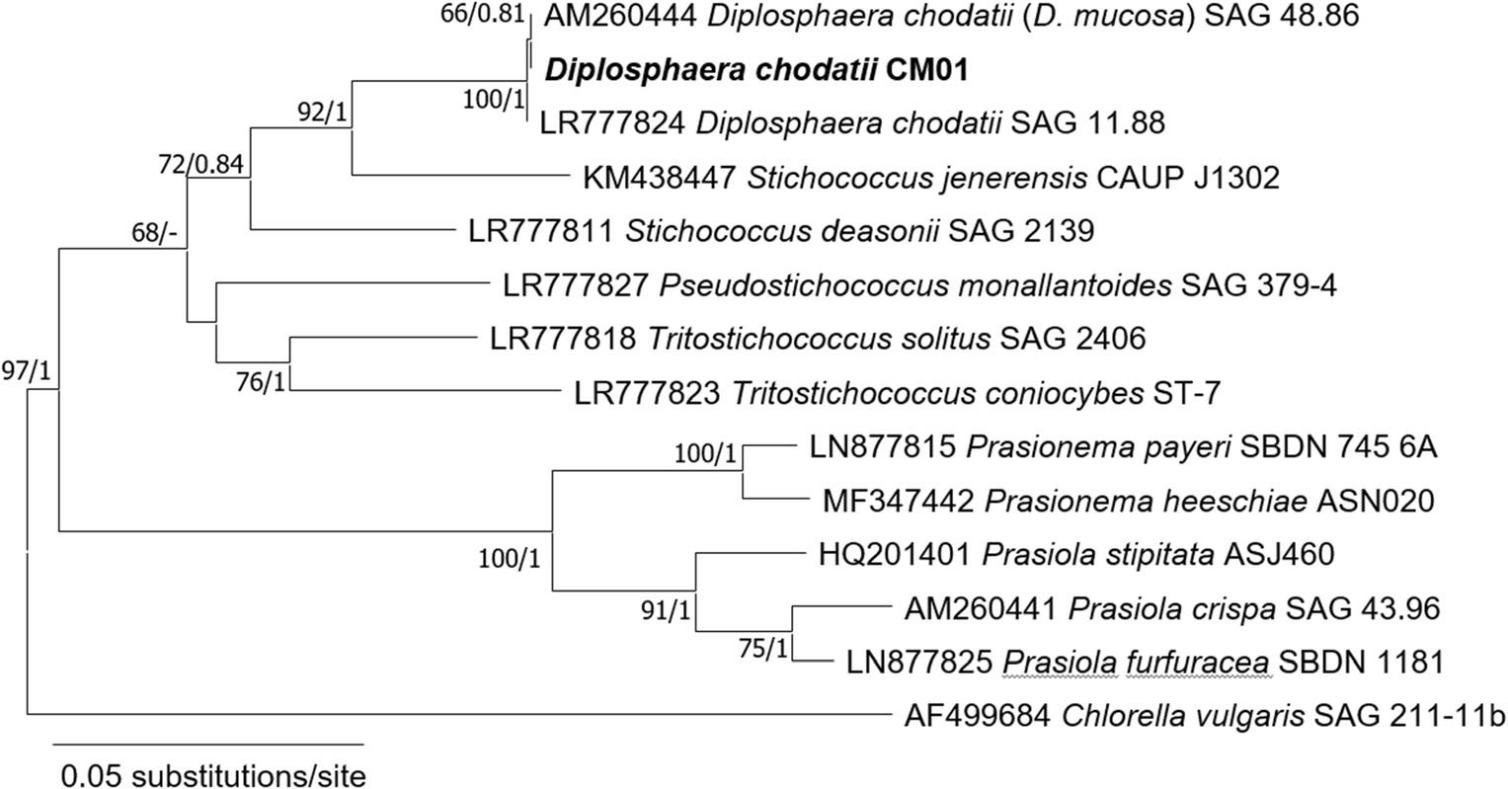
Maximum-likelihood tree based on *rbc*L gene sequences of investigated bark tree isolate CM01 from the botanical garden of Innsbruck (bold). Maximum-likelihood tree was constructed in MEGA X (Kumar et al. 2018) using the evolutionary model GTR + G + I, the bootstrap support was calculated with 1000 replicates; *Chlorella vulgaris* served as outgroup. Bayesian analyses were conducted in MrBayes 3.2.2 (Ronquist and Huelsenbeck 2003) using the evolutionary model GTR + G + I with 1,000,000 generations. Support values are given at the nodes; ML bootstrap support < 50% and Bayesian probability below 0.8 are not shown. The scale bar corresponds to 0.05 substitutions per site

The newly isolated algal strain CM01 was identified as *Diplosphaera chodatii* within the class Trebouxiophyceae using the *rbc*L gene. The *rbc*L sequence was identical to the authentic strain SAG 11.88 (Fig. 2) and similar to *Stichococcus jenerensis* (KM438447). Cells of *D. chodatii* were unicellular, differed in their shape from spherical to ellipsoidal and varied in size about 3.5–7.0 μm in length and 2.5–4.0 μm in width (Fig. 1c, d), with a cup-shaped parietal chloroplast (Fig. 1d). The cells were either singular or formed dyads (two cells were connected, Fig. 1c, d). Some, but not all, cells were surrounded by light microscopically visible mucilage. No cell packets were observed.

**Fig. 3:**
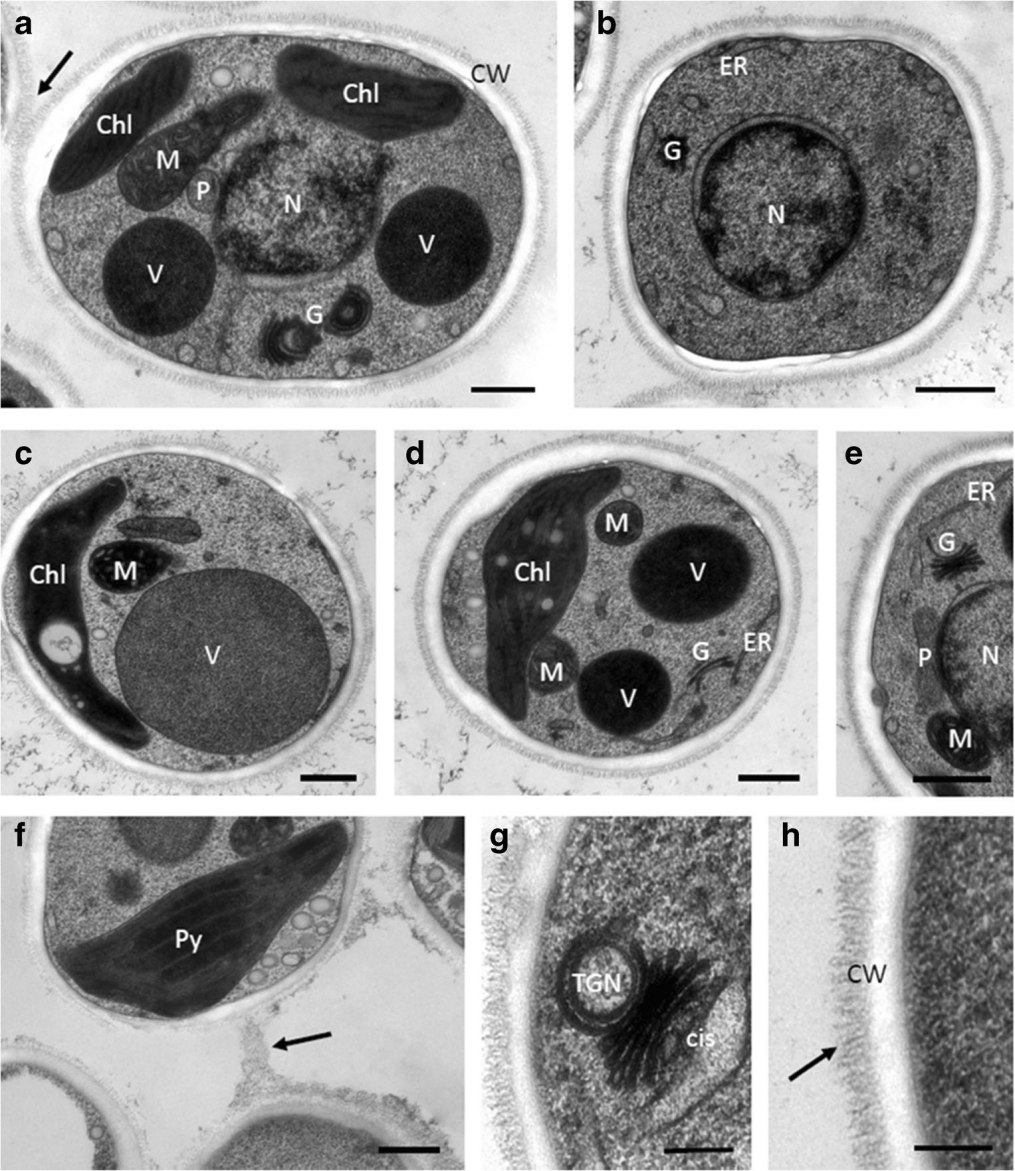
**T**ransmission electron micrographs of *Diplosphaera chodatii* CM01. **a** Longitudinal section in median plane with two chloroplast lobes, mitochondrion, Golgi bodies, vacuoles and peroxisome, **b** transversal section in median plane, **c** transversal section showing mitochondrion, large vacuole and parietal chloroplast, **d** transversal section with electron-dense vacuoles, parietal chloroplast, endoplasmatic reticulum (ER), **e** median section with central nucleus and peroxisome, Golgi body, mitochondrion and ER, **f** detail with two cells connected by mucilage (arrow), in the chloroplast a pyrenoid is visible, **g** detail of Golgi body with trans Golgi network (TGN) and cis side clearly visible, **h** detail of the cell wall with smooth inner layer and fuzzy hair-like outer polysaccharide layer (arrow). Chl, chloroplast; CW, cell wall; ER, endoplasmatic reticulum; G, Golgi body; M, mitochondrion; P, peroxisome; Py, pyrenoid; scale bars **a**–**f** 500 nm, **g**–**h** 250 nm

The ultrastructure of *D. chodatii* showed the lobed parietal chloroplast with regularly arranged thylakoid membranes. No starch grains could be detected in the chloroplast (Fig. 3a–f), but sometimes ‘naked pyrenoids’ were visible (Fig. 3f). When cells were longitudinally sectioned, a central nucleus, mitochondria, Golgi bodies, a peroxisome, and electron-dense vacuoles were visible (Fig. 3a). Transversally sectioned cells showed the nucleus (Fig. 3b) or more towards the cell periphery the parietal chloroplast, and large vacuoles of varying electron density were observed (Fig. 3c, d). The mitochondria were large and dense; a peroxisome was observed between the nucleus and mitochondrion (Fig. 3c). Several Golgi bodies were detected (Fig. 3d, e) and the pairwise arranged vacuoles appeared electron dense (Fig. 3a, d). The cell wall was bilayered; an inner smooth area was covered by an up to ~150-nm-thick layer of fibrillary polysaccharides with a fuzzy hair-like structure, evident in every investigated cell (Fig. 3a–h). A detail of this bilayered cell wall is illustrated in Fig. 3h. Sometimes cells were connected with this fibrillary layer (Fig. 3a, arrow) or with mucilage attached outside of this layer (Fig. 3f, arrow). Apparently, the hair-like structures allow the cells to stick together on to their surface. The Golgi bodies were very prominent and contained up to 5 cisternae; a cis-side and a trans Golgi network (TGN) was clearly visible (Fig. 3g).

### Light requirements on photosynthetic performance and growth

**Fig. 4:**
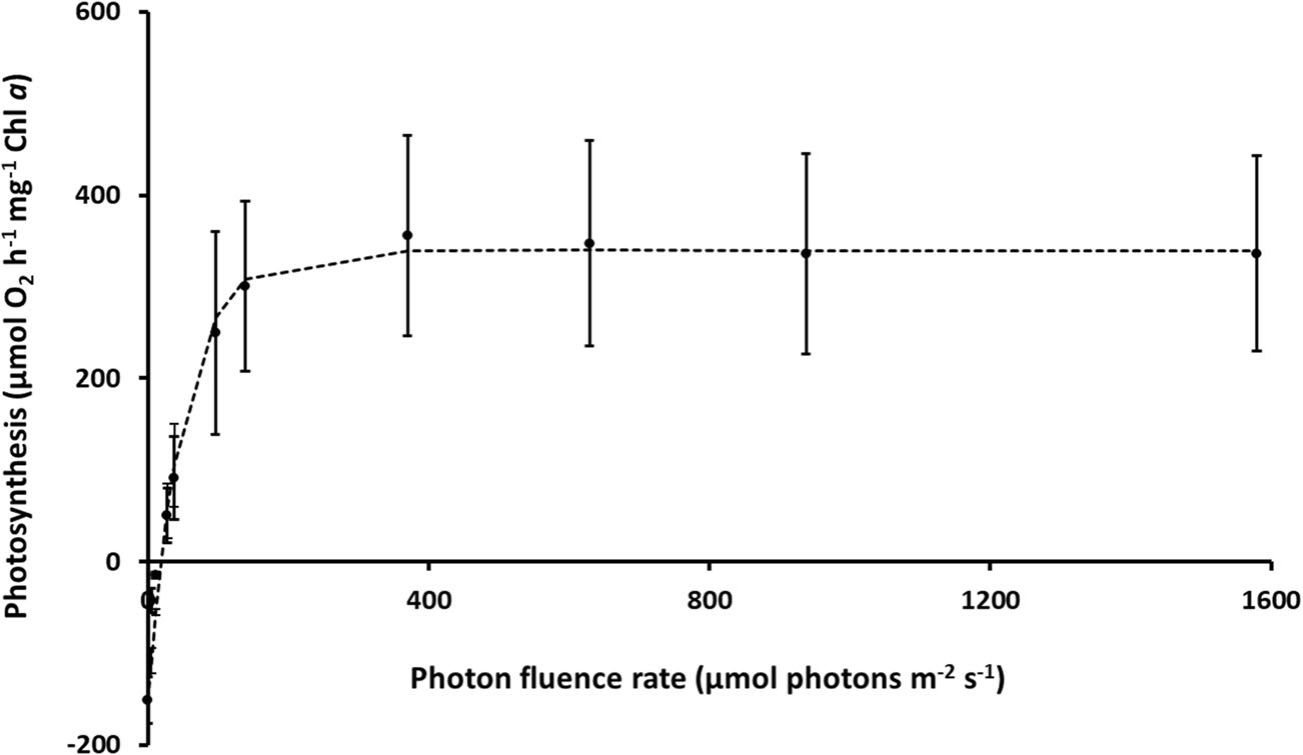
The effect of increasing photon flux densities (in μmol photons m^−2^ s^−1^) on photosynthetic oxygen evolution of log-phase *Diplosphaera chodatii* CM01 (*n*=4, mean value ± SD). The dotted line represents a fitted curve after Walsby (1997)

Increasing photon flux densities (PFDs) stimulated the photosynthetic oxygen production (Fig. 4). While the respiration rate amounted − 153 μmol O_2_ h^−1^ mg^−1^ Chl *a*, maximum photosynthetic rate (P_max_) in the light-saturated range was 340 μmol O_2_ h^−1^ mg^−1^ Chl *a*. *Diplosphaera chodatii* exhibited an I_c_ value of 16.6 μmol photons m^−2^ s^−1^ and an I_k_ value of 50.6 μmol photons m^−2^ s^−1^. No photoinhibition could be detected under the highest photon flux density (1580 μmol photons m^−2^ s^−1^) during short time exposure.

After 4 days at 846 μmol photons m^−2^ s^−1^, pre-cultures of *D. chodatii* bleached and hence no chlorophyll fluorescence was detectable. Therefore, permanent light exposure to 846 μmol photons m^−2^ s^−1^ is lethal for growth. Color intensity of the green cultures changed in dependence on photon flux densities applied (results not shown). At 35 μmol photons m^−2^ s^−1^, all replicates were dark green, while exposure for three days to 364 or 581 μmol photons m^−2^ s^−1^ was accompanied by a conspicuous change to bright green.

**Fig. 5:**
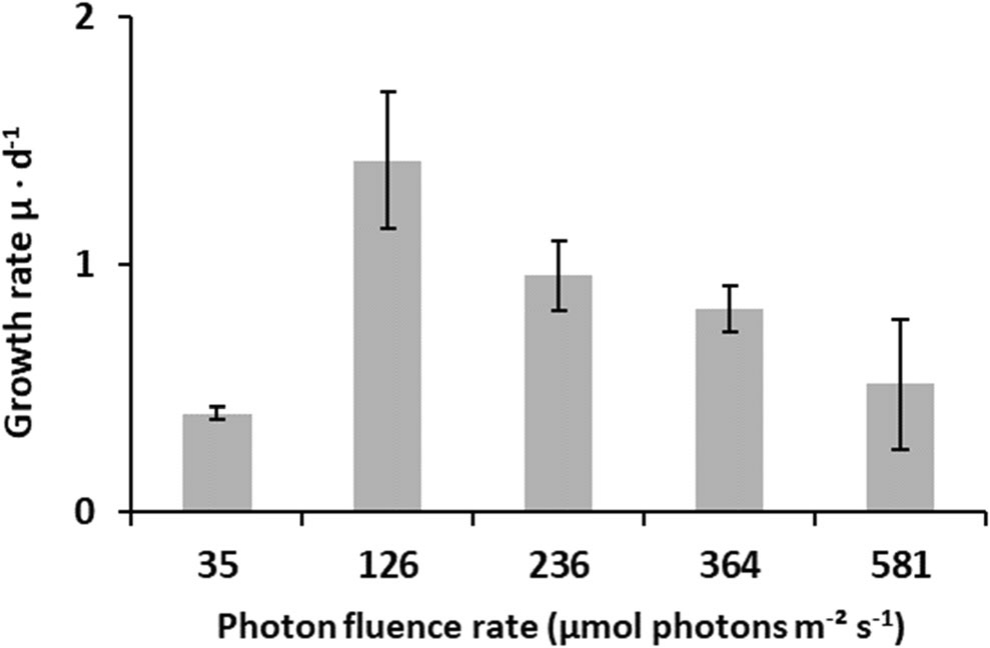
Relationship between the growth rate (μ d^−1^) and increasing photon flux density (in μmol photons m^−2^ s^−1^) in *Diplosphaera chodatii* CM01. Growth rates were calculated after Heuser (2003) (*n*=5, mean value ± SD). Only the data from day 1 to day 14 were considered. All measurements were done at 20 °C ± 1 °C. Significances among the treatments were calculated by one-way ANOVA (*p*<0.05). Different small letters represent significant differences among the photon flux density as revealed by Tukey’s post hoc test

The growth rates (μ) of *D. chodatii* showed significant differences depending on the photon flux density (PFD) (Fig. 5). Lowest growth rate was observed at 35 μmol photons m^−2^ s^−1^ and at 581 μmol photons m^−2^ s^−1^. The highest growth rate (1.42 ± 0.28 μ day^−1^) was achieved at 126 μmol photons m^−2^ s^−1^, which was about 3.5 times higher than at 35 and 581 μmol photons m^−2^ s^−1^. Cultures exposed to 236 and 362 μmol photons m^−2^ s^−1^ exhibited intermediate growth rates (68% and 59% of the maximum, respectively). The maximum growth rate under optimum conditions (126 μmol photons m^−2^ s^−1^) was reached after 5 days treatment, while those at 236 μmol photons m^−2^ s^−1^ and 362 μmol photons m^−2^ s^−1^ after 7 days (data not shown).

### Effects of controlled dehydration and rehydration on effective quantum yield

**Fig. 6:**
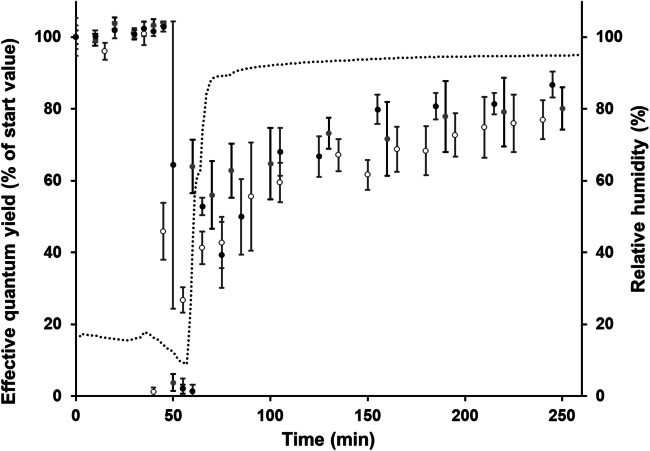
The effect of controlled desiccation and rehydration on the effective quantum yield (Y(II)) of PSII in *Diplosphaera chodatii* CM01 measured with a PAM2500. Silica gel was used to create a relative humidity of ~10%. All measurements were done at 22 °C ± 1 °C. Effective quantum yield values were standardized to the starting Y(II) to 100% for better comparison. Black, gray, and white circles represent biological replicates (*n*=4 ± SD). The dotted line shows the curve of the relative humidity in the box

*Diplosphaera chodatii* was tested for desiccation tolerance by using non-invasive PAM measurements according to Karsten et al. (2014). The effective quantum yield was constant during dehydration for about 45 min in desiccating atmosphere (RH of 10%) (Fig. 6). After 55 min, the algae were rehydrated and transferred to RH of 95%. The effective quantum yield of the algae increased to approximately 85% of the original Yield (II) value within 190 min after recovery.

**Fig. 7:**
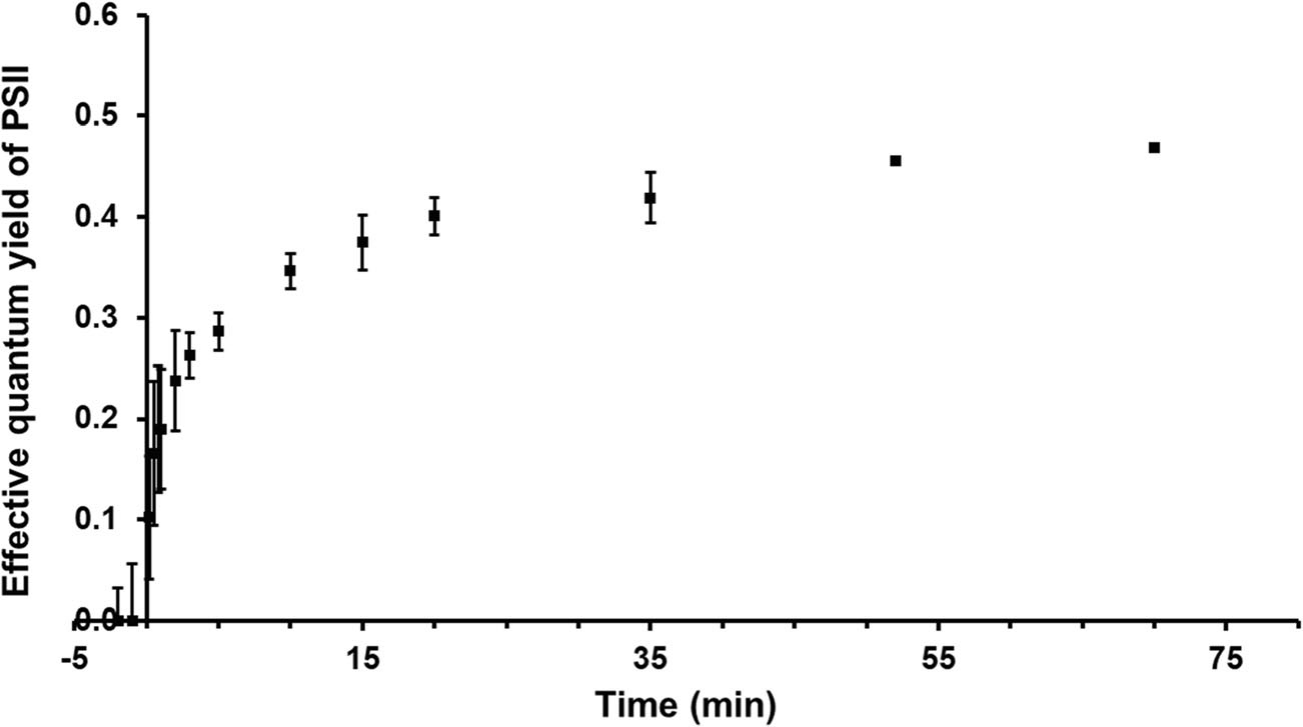
**K**inetics of photosynthetic activity of a natural biofilm of *Diplosphaera chodatii* taken from the cypress tree bark after rewetting with tap water (*n* = 3 ± SD). Effective quantum yield of PSII was measured with a PAM2500

In addition, the photosynthetic activity of a natural biofilm of *D. chodatii* taken at noon from the cypress tree bark was followed after rewetting with tap water. The effective quantum yield of the naturally air-dried cells was zero. However, already a few seconds after rewetting, the effective quantum yield exhibited a value of 0.2 (Fig. 7). Within the following 30 min, the effective quantum yield increased to > 0.4, indicating a very quick recovery of photosynthetic activity of the algal cells after contact with liquid water.

### Screening for MAAs and osmoprotectans

**Fig. 8:**
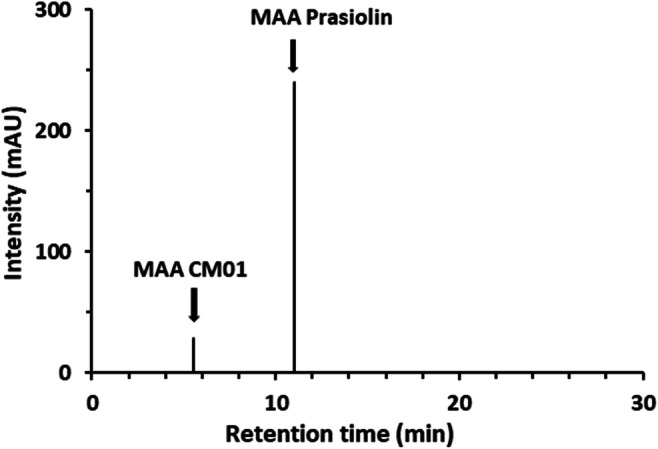
**H**PLC chromatogram of extracts of *Diplosphaera chodatii* CM01 and prasiolin extracted from *Prasiola calophylla.* Prasiolin eluted at 10.9 min and the extract of CM01 showed a MAA eluting at 5.3 min

**Fig. 9:**
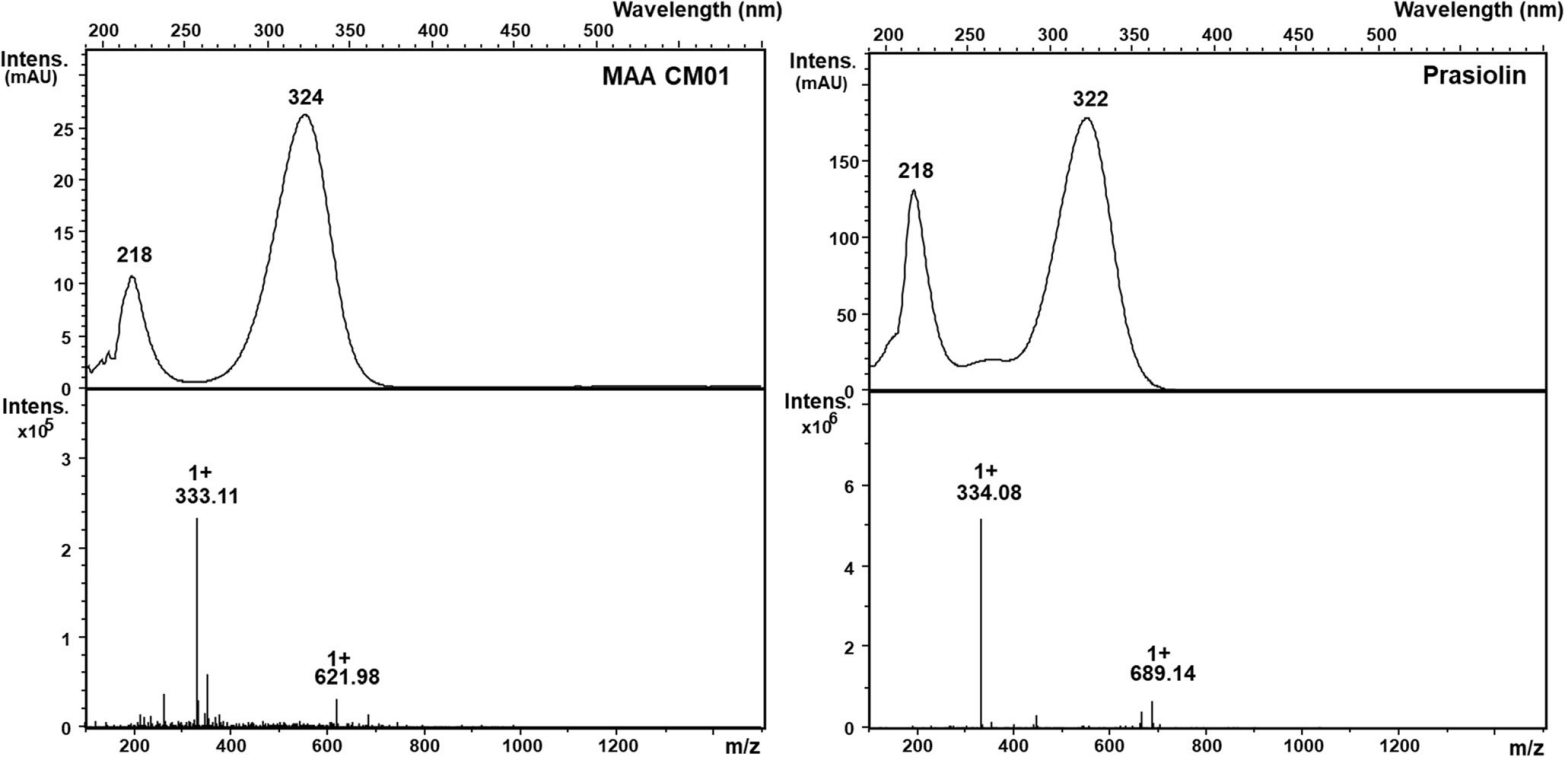
UV and ESI-MS spectra of the UVA-absorbing compound in *Diplosphaera chodatii* CM01 (left) and of prasiolin extracted from *Prasiola calophylla* (right). ESI-MS was measured in positive mode

HPLC-MS analysis was performed to screen *D. chodatii* for desiccation and UV protective substances. In the sample, a peak with a retention time of 5.3 min was visible (Fig. 8), which exhibited an absorption maximum of 324 nm and the molecular mass of 332 g mol^−1^ (Fig. 9). This compound could unambiguously be discriminated from the MAA prasiolin (*Prasiola calophylla* served as reference, Figs. 8 and 9), which exhibited a retention time of 10.9 min with this method, an absorption maximum of 322 nm, and a molecular mass of 333 g mol^−1^.

**Fig. 10:**
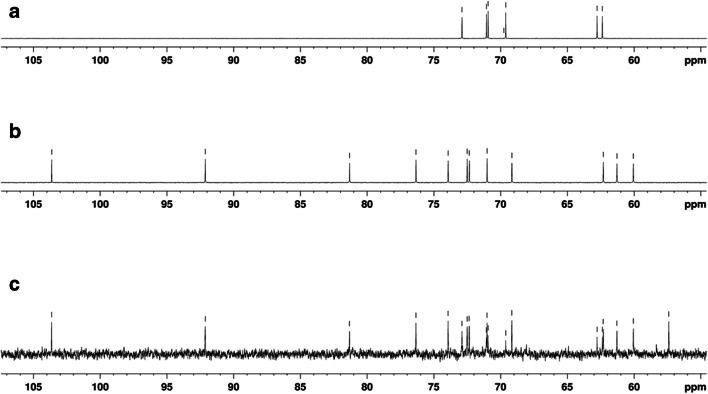
^**1**3^C NMR-spectra of *D. chodatii* CM01 in D_2_O*.* **a** Sorbitol, **b** sucrose, **c** total spectrum of an algal extract

Furthermore, the extract of *D. chodatii* (Fig. 10c) contained sorbitol (Fig. 10a) and sucrose (Fig. 10b) as shown in the ^13^C NMR spectrum (for more details, see Table 1).

**Table 1:** ^13^C NMR chemical shifts for sucrose and sorbitol in extracts of *Diplosphaera chodatii* CM01 (ppm)

Sucrose	Sorbitol
60.1	62.4
61.3	62.8
62.3	69.6
69.2	70.9
71.0	71.1
72.3	72.9
72.5	
73.9	
76.3	
81.3	
92.1	
103.6	

## Discussion

*Diplosphaera chodatii* is characterized by a high diversity in cell shape, habitat preference, and lifeform. For example, this taxon is well studied as a photobiont in lichens (Zhang and Wei 2011; Fontaine et al. 2013) since it is reported to be the most common algal symbiont in the Verrucariaceae (Thüs et al. 2011). In the present study, a free-living *D. chodatii* isolated from a tree bark was characterized for the first time for various traits potentially essential for a terrestrial lifestyle.

The new strain CM01 is characterized by its variability of the cell shape, similar as described for other *D. chodatii* isolates (Fontaine et al. 2013; Raabová et al. 2016). These findings underline the high phenotypic plasticity of *D. chodatii*. This trait has also been observed in other unicellular members of the Trebouxiophyceae such as *Chloroidium* or *Chlorella*-like species (see Darienko et al. (2010) and references therein) which may be interpreted as adaptive mechanism to different environmental conditions. In a recent study, Pröschold and Darienko (2020) compare the length to width ratios of many Trebouxiophyceae, and report values slightly above 1 for different *Diplosphaera* spp. including SAG 11.88, which is the closest related taxon to the here investigated CM01. Our observations gave higher length to width ratios between 1.4 and 1.7.

To the best of our knowledge, we are not aware of transmission electron micrographs of the genus *Diplosphaera*. However, there are some reports from the closely related *Stichococcus*, which exhibited smooth cell wall surfaces (Pickett-Heaps 1974; Yamamoto et al. 2016). Yamamoto et al. (2016) showed that dividing *Stichococcus* cells are surrounded by a mother cell wall that is then disrupted, being a distinct structure from what is observed here. Therefore, our data of the outer cell wall layer in *D. chodatii* with a fuzzy hair-like appearance are novel, and likely support the lifestyle of this epiphytic alga, giving a structural basis for adhesion. However, currently we can only hypothesize on the biopolymer composition of the fuzzy outer cell wall layer, which could be composed of acidic polysaccharides containing rhamnose and galacturonic acid, because these compounds give a similar appearance and are common in related genera like *Chlorella* sp., or *Chloroidium* (Alhattab et al. 2019). Occasionally larger mucilage layers could be observed between the cells (Fig. 3f), but their biochemistry remains also enigmatic. The inner smooth cell wall layer likely contains cellulose, glucomannans, xylans, and even algaenans as these biopolymers are also found in related genera of the Trebouxiophyceae (see Table 1 in Alhattab et al. 2019). Moreover, Trebouxiophyceae may contain, besides the standard cell wall polysaccharides, also compounds usually only common in fungi like galactofurans, and gluco- and galactosamines (Alhattab et al. 2019). The occurrence of numerous Golgi bodies points towards an active polysaccharide synthesis, and structures like the TGN were observed, which are difficult to preserve for TEM (Fig. 3g). The vacuoles had a high electron density, suggesting that they contained substances reacting with osmium used during the freeze substitution procedure. Similar vacuoles with electron-dense content were found in various green algae, including, e.g., *Chlorella vulgaris*, *Acutodesmus obliquus*, and *Parachlorella kessleri* (Shebanova et al. 2017). The appearance of the electron-dense content as well as the low number of vacuoles was similar as observed in this study. Overall, the appearance of the cytoplasm is very dense additionally providing evidence for a high fixation quality.

### Light requirements for photosynthesis and growth and adaptation to UVR

*Diplosphaera chodatii* showed all features of low light requirements for photosynthesis due to low I_c_ and I_k_ values together with lack of photoinhibition even at the highest PFD tested (1580 μmol photons m^−2^ s^−1^), indicating a high tolerance to strong solar radiation, at least for short time periods. This suggests flexible acclimation mechanisms of the photosynthetic apparatus (photophysiological plasticity) to highly variable light conditions, ranging from shading clouds to direct sun light and, further, diurnal and seasonal variations. During summer, the PFD at the bark surface in the Botanical Garden ranged from 80 to 800 μmol photons m^−2^ s^−1^ (own results, not shown) explaining the abundant occurrence of *D. chodatii*. Such high photophysiological plasticity was also shown in streptophytic *Zygnema* sp. and *Klebsormidium crenulatum* but seems not to be a common trait of all aeroterrestrial green algae (Karsten et al. 2010a; Herburger et al. 2015).

For growth the newly isolated strain CM01 preferred low-light conditions as the maximum growth rate was detected at 126 μmol photons m^−2^ s^−1^. However, the alga had the ability to grow over a broad light range from 35 to 581 μmol photons m^−2^ s^−1^ reflecting the environmental conditions in terrestrial habitats, which are characterized by strongly fluctuating light conditions. Nevertheless, a lethal growth limit at 846 μmol photons m^−2^ s^−1^ was detected for *D. chodatii* after long-term treatment of 4 days.

Low-light requirements for growth combined with no signs of photoinhibition even at higher PFDs were also detected in other terrestrial microalgae, for example, in an alpine isolate of *Chlorella vulgaris* (Aigner et al. 2020). Considering the natural light conditions at a tree bark, this terrestrial microhabitat is from an ecophysiological point of view challenging for microalgae, as the bark is exposed to both strong diurnal and seasonal light fluctuations. The data presented demonstrate that *D. chodatii* exhibits a rather broad tolerance for the prevailing light conditions as it can efficiently photosynthesize between low and high PFDs and still shows growths between 35 μmol photons m^−2^ s^−1^ and < 581 μmol photons m^−2^ s^−1^. In addition, *D. chodatii* exposed to 35 μmol photons m^−2^ s^−1^ revealed dark green color in comparison to cells kept under higher PFDs, which turned to light green. Therefore, *D. chodatii* potentially follows the same acclimation strategy to low light conditions as many marine phytoplankton species of the *Chlorella*-type (Steemann-Nielsen and Jørgensen 1968). These algae increase and decrease their chlorophyll content under low and high light conditions, respectively, in order to optimize their photosynthetic productivity. This mechanism seems to be an additional important acclimation process of *D. chodatii* on tree barks under fluctuating light conditions.

The UV-protective MAAs in terrestrial algae can be used for chemotaxonomic assignments (Hotter et al. 2018) as these compounds are widely distributed in members of the green algal class Trebouxiophyceae (Karsten et al. 2005). In their study, all investigated members of the *Prasiola* clade showed the same MAA termed “prasiolin” (absorption maximum 322 nm) with an absorption maximum similar, but not identical to the putative MAA of the strain CM01 at 324 nm (Hartmann et al. 2016). In combination with the molecular weight of the MAA found in CM01 (332 g mol^−1^), which does not match with any previously published data on MAAs, this MAA is novel. The structure of the new MAA probably differs by one hydrogen atom from that of prasiolin, because the measured molecular mass was only 332 g mol^−1^ instead of 333 g mol^−1^ from prasiolin. Additionally, prasiolin and the new MAA exhibited different retention times (10.9 vs. 5.3 min) during the HPLC analysis.

Controlled UVR-exposure experiments led to a strong and dose-depending biosynthesis and accumulation of prasiolin in numerous terrestrial Trebouxiophyceae, thus supporting its function as an UV sunscreen (Karsten et al. 2007b). The accumulation of MAAs in *Stichococcus* sp. and *Chlorella luteo-viridis* was accompanied by a reduced UV sensitivity of photosynthesis and growth, which well reflects the conspicuous ecological success of many Trebouxiophycean taxa in harsh terrestrial environments (Karsten et al. 2007b).

### Desiccation

Several studies, like Karsten et al. (2014), showed that desiccation tolerance strongly correlates with the cell organization, as cell associations in an aggregate, colony, or biofilm enable the algae to hold water in the extracellular matrix. Although the microscopic observations of *D. chodatii* did not show any cell aggregate formation, the fuzzy hair-like structures on the outer layer of the cell wall are assumed to function like an adhesive tape, thereby indicating preliminary stages of biofilm formation. This is supported by the detected mucous secretion found in vitro in CM01 which may result in biofilm formation in vivo and could reduce stress exposure at least for parts of the population (Karsten et al. 2007b). Therefore, it would be highly interesting to further characterize the biochemical composition of the cell wall of the newly isolated strain.

*Diplosphaera chodatii* revealed desiccation tolerance when exposed to RH of 10%. Within a relatively short recovery period of 190 min, 85% of the original photosynthetic activity was reached indicating the ability to tolerate desiccation stress for a short time period. This rapid recovery after dehydration is considered characteristic ecophysiological trait of terrestrial microalgae as shown for a terrestrial strain of *Chlorella vulgaris* (Trebouxiophyceae; Aigner et al. 2020), terrestrial strains of *Tetradesmus* sp. (Chlorophyceae; (Terlova et al. 2021) and for the streptophyte *Klebsormidium crenulatum* (Karsten et al. (2010a). Under natural conditions, green algal biofilms are often inactive but vital. After precipitation events (rain, fog, dew etc.), such biofilms get quickly reactivated, i.e., they photosynthesize until water loss is getting too high to support further physiological activity (Häubner et al. 2006). In addition, frequent desiccation stress events trigger various protective mechanisms, such as the biosynthesis and accumulation of low-molecular-weight osmoprotectants that increase water-holding capacity of the algal cells (Farrant 2000; Gustavs et al. 2011; Fernández-Marín et al. 2016).

Based on the data from HPLC and NMR spectroscopy, *D. chodatii* synthesized and accumulated sucrose and sorbitol as main organic osmolytes and hence as protectants against desiccation stress. Sorbitol is quite common in members of the Trebouxiophyceae, e.g., in *Diplosphaera*-related *Stichococcus* sp. (Gustavs et al. 2011; Hotter et al. 2018). Sucrose was also found in terrestrial *Chlorella vulgaris* (Aigner et al. 2020). As a common metabolite in *Klebsormidium* species and higher plants, sucrose is often associated to cold acclimation (Nagao et al. 2008; Nagao and Uemura 2012; Holzinger and Pichrtová 2016).

### Conclusion

The present study provides a detailed overview of the ecophysiological, biochemical, and ultrastructural traits of the terrestrial free-living alga *D. chodatii* isolated from a tree bark. Transmission electron microscopy showed polysaccharides with a fuzzy-hair-like structure in the outer layer of the cell wall. Together with the occasional occurrence of mucilage, this structure could be regarded as a kind of precursor for biofilm formation. Moreover, the discovered structure may have a capacity in holding the cells together as well as in attaching cells to their surface. This may be an adaptation to fluctuating water availability, as biofilm formation is known to reduce desiccation or radiation stress.

Furthermore, *D. chodatii* CM01 exhibited a pronounced desiccation tolerance, which can be explained by the biochemical capability to synthesize and accumulate sorbitol and sucrose as protective compounds. In addition, *Diplosphaera* showed low light requirements for photosynthesis along with no signs of photoinhibition pointing to high photophysiological plasticity, and a previously unknown putative MAA with a molecular mass of 332 g mol^−1^ and an absorption maximum at 324 nm was discovered. The combination of all these adaptive traits well explains the obvious ecological success of *D. chodatii* as bark tree alga.

## Supplementary Information

ESM 1 (DOCX 15 kb)
