# Supplementary material for: Ecophysiological, morphological, and biochemical traits of free-living Diplosphaera chodatii (Trebouxiophyceae) reveal adaptation to harsh environmental conditions
Source: Protoplasma. 2021 Feb 7;258(6):1187–99. doi: 10.1007/s00709-021-01620-6 (PMC8523416; doi:10.1007/s00709-021-01620-6)
Supplement: Supplementary file 1 — (DOCX 15 kb) [file 709_2021_1620_MOESM1_ESM.docx]

Suppl. Table 1: Initial weight of desiccants resulted in different relative humidity inside the polystyrol box.

| **Desiccant** | **Initial weight** | **approx. reached relative humidity** |
| --- | --- | --- |
| sat. Potassium chloride | 33g/100 mL Aqua_dest._ | 85% |
| Lithium chloride | 23 g/ 100 mL Aqua_dest._  30 g/ 100 mL Aqua_dest._  40 g/ 100 mL Aqua_dest._  50 g/ 100 mL Aqua_dest._ | 69%  57%  37%  31% |
| freshly activated Silica gel | 100 g | 10% |
